# Addendum: Pesce et al. Irisin and Autophagy: First Update. *Int. J. Mol. Sci.* 2020, *21*, 7587

**DOI:** 10.3390/ijms22105117

**Published:** 2021-05-12

**Authors:** Mirko Pesce, Patrizia Ballerini, Teresa Paolucci, Iris Puca, Mohammad Hosein Farzaei, Antonia Patruno

**Affiliations:** 1 Department of Medicine and Aging Sciences, University G. d’Annunzio, 66100 Chieti, Italy; antonia.patruno@unich.it; 2 Department of Neurosciences, Imaging and Clinical Sciences, University G. d’Annunzio, 66100 Chieti, Italy; 3 Department of Oral, Medical and Biotechnological Sciences, University G. d’Annunzio, 66100 Chieti, Italy; teresa.paolucci@uniroma1.it; 4 Sport Academy SSD, 65010 Pescara, Italy; pucairis@gmail.com; 5 Pharmaceutical Sciences Research Center, Health Institute, Kermanshah University of Medical Sciences, Kermanshah 67146, Iran; mh.farzaei@gmail.com

We did not receive the copyright for Figure 2 in our published paper [1]. Now, we have received it from the correspondence author Ewa Pocheć [2]. Therefore, we would like to announce this addendum.

This addendum does not cause any changes to the results and conclusions in the original published paper.

## References

[1] Pesce M., Ballerini P., Paolucci T., Puca I., Farzaei M.H., Patruno A. (2020). Irisin and Autophagy: First Update. Int. J. Mol. Sci..

[2] Korta P., Pocheć E., Mazur-Biały A. (2019). Irisin as a Multifunctional Protein: Implications for Health and Certain Diseases. Medicina.

